# Assessing pyridoxine adjuvant therapy effects on blood glucose levels in type 2 diabetes: A randomized clinical trial

**DOI:** 10.25122/jml-2023-0178

**Published:** 2023-10

**Authors:** Moatamad Hanoon Dawood, Manal Khalid Abdulridha, Hayder Saadoon Qasim

**Affiliations:** 1 Department of Clinical Pharmacy, College of Pharmacy, Mustansiriyah University, Baghdad, Iraq; 2 Department of Medicine, College of Medicine, Maysan University, Maysan, Iraq

**Keywords:** pyridoxine adjuvant therapy, type 2 diabetes, glycemic control, vitamin, metformin

## Abstract

Pyridoxal-5-phosphate (PLP) is the bioactive derivative of vitamin B6, functioning as a coenzyme in over 150 metabolic pathways. Insufficient PLP levels could be associated with the onset and progression of diabetes. This study aimed to assess the effects of pyridoxine adjuvant treatment on blood glucose levels in patients with type 2 diabetes mellitus (T2DM). This interventional, randomized, open-label study was conducted in the Mesan Governorate, with participants from the Mesan Center for Diabetes and Endocrinology as the study population. This study included patients newly diagnosed with T2DM. Patients were randomized into three groups: Group 1, the control group, treated with non-pharmacological therapy (lifestyle modification) (n=20); Group 2, treated with Metformin 500 mg/day in addition to non-pharmacological therapy (lifestyle modification) (n=20). Group 3 was treated with Metformin 500 mg/day plus vitamin B6 300 mg/day in addition to non-pharmacological therapy (lifestyle modification) (n=68). The findings revealed a considerably favorable impact of pyridoxine adjuvant treatment with Metformin on blood glucose levels and other study variables. Compared to the patients in the control group G1, the reductions in fasting plasma glucose (FPG) and glycated hemoglobin (HbA1c) were statistically significant in groups G2 and G3 after a 4-week treatment period. Similar results were observed for fasting serum insulin and homeostasis model assessment of insulin resistance (HOMA-IR) levels, with a significant decrease in groups G2 and G3 (p<0.05). Furthermore, the reductions in indoleamine 2,3-dioxygenase levels were also significantly higher in groups G2 and G3 at the end of the 4-week treatment period (-14.48% *vs* -21.16%) (p<0.05). Adding pyridoxine adjuvant therapy to Metformin treatment could effectively improve the blood glucose levels of patients with T2DM.

## INTRODUCTION

Type 2 diabetes mellitus (T2DM) can cause persistent impairment, dysfunction, and failure in multiple organs. While the initial symptoms of T2DM may include weight loss, frequent urination, thirst, hunger, and vision impairment, it is essential to note that this disorder also has long-term effects [1-4]. These long-term effects might involve the gradual development of specific complications such as cardiovascular disease (CVD) and retinal degeneration, which can lead to vision loss and kidney failure. Renal failure and neuropathy are correlated with an increased likelihood of developing foot ulcers, Charcot joints, and signs and symptoms of autonomic dysfunction, including sexual dysfunction [5].

The factors contributing to the prevalence of T2DM include growing urbanization, limited engagement in physical exercise, a lack of activity, and obesity. While it may not be possible to modify all causes, a significant portion may be addressed by appropriate lifestyle habits, avoiding drug misuse, and implementing screening measures to detect potential issues [6-8].

According to Salvo *et al*. [11], pyridoxal-5-phosphate (PLP), the active component of vitamin B6, functions as a coenzyme in approximately 150 different enzymatic reactions, primarily involved in the processing of carbohydrates, lipids, and proteins, in addition to participating in neural functions that are modulated by the synthesis and degradation of vitamin B6. The PLP molecule serves as a mediator by inhibiting the formation of reactive oxygen species (ROS), as demonstrated by Ehrenshaft *et al*. in 1999 [9]. Additionally, it impedes the generation of advanced glycation end products (AGEs), which are believed to be linked to aging and diabetes and are known to possess genotoxic properties, as reported by Booth *et al*. [10]. In contrast to microbes, mammals are deficient in pyridoxal-5-phosphate production. As a result, they depend on the vitamin B6 recycling pathways found in their diet, including pyridoxal, pyridoxamine, and pyridoxine [11]. The cytoplasmic recycling of pyridoxal, pyridoxamine, and pyridoxine through vitamin-5-phosphorylate is facilitated by pyridoxal kinase [11]. Hellman and Mooney established a correlation between medical disorders of significant importance, such as vitamin B6 deficiency, and various health concerns, including autism, mental illness, dementia, Parkinson's disease, seizures, developmental delays, diabetes, and malignancy [12]. Additionally, there was a correlation between diabetes and vitamin B6 [13]. The causal relationship between low PLP levels and diabetes remains unclear. It has been suggested that inadequate levels of PLP may lead to the development of diabetes [14]. However, Okada *et al*. presented contrasting findings, indicating that diabetes may cause a reduction in PLP levels [15]. Despite incomplete comprehension of the underlying cellular and molecular mechanisms responsible for the beneficial impact on diabetes pathology and its related consequences, various studies have reported the beneficial effects of B6 therapy [16, 17]. PLP deficiency can affect diabetes through multiple mechanisms. Pyridoxal-5-phosphate is a crucial component of various enzymes that aid this process [18]. It can affect the pathway that transforms tryptophan into niacin, as demonstrated by Oxenkrug *et al*. [19]. Impairment of this pathway produces metabolites that reduce the bioactivity of insulin and lead to insulin resistance, a symptom of T2DM [20]. Pyridoxal-5-phosphate can potentially affect insulin resistance by regulating the expression of genes associated with adipogenesis [21]. The escalation of homocysteine concentration due to the decay of co-enzyme-dependent enzymes such as cystathionine-β-synthase (CBS) and cystathionine gamma-lyase (CGL), which are dependent on pyridoxal-5-phosphate, can lead to insulin resistance [22].

The present study aimed to assess the effect of pyridoxine adjunctive treatment on the glycemic status of individuals diagnosed with T2DM.

## MATERIAL AND METHODS

### Study design and setting

This single-center, interventional, randomized, controlled, open-label study was conducted between the first of November 2022 and the end of March 2023 in Mesan Governorate-Iraq at the Mesan Center for Diabetes and Endocrinology.

### Participants

Participants were recruited from the Mesan Center for Diabetes and Endocrinology. Inclusion criteria comprised individuals who were newly diagnosed with type 2 diabetes mellitus, aged 30 years or older, and with anglycated hemoglobin (HbA1c) level equal to or less than 7.5%. Exclusion criteria included individuals with type 1 diabetes mellitus, those who had been previously diagnosed with type 2 diabetes mellitus and received diabetes treatment, individuals with concomitant chronic diseases such as rheumatoid arthritis, anemia, bronchial asthma, or those taking anti-tuberculosis (anti-TB) or anti-epileptic medications. Pregnant women or those using female oral contraceptive drugs, individuals taking vitamin or mineral supplements, those with a recent history of acute infection within the previous two weeks, and alcoholic patients were also excluded from the study.

### Sample size

The sample size was determined and calculated using G*Power 3.1.9.7 (RRID: SCR 013726). The minimum sample size of the study was 109 participants, with an effect size of 0.33, 95% statistical power, and a two-tailed alpha of 0.05, resulting in a 95% confidence interval (f). Approximately 129 individuals were screened for potential enrollment; however, only 108 met the established inclusion criteria, with the remaining individuals being excluded from the study.

### Study groups

Participants were randomly allocated to one of the following three groups. The researchers used predetermined lists stratified by sex and age to consecutively assign code numbers to study participants.

**Group 1 (Control) (n=20):** This group included newly diagnosed patients with T2DM who received non-pharmacological therapy. The initial assessment and patient education were administered by a physician specializing in endocrinology. The recommended lifestyle modifications included encouraging patients to engage in at least two and a half hours of physical activity per week at a moderate intensity level or, dedicating one hour and 15 minutes per week to high-intensity exercise. Participants were also advised to gradually reduce their weight to achieve a healthy body mass index. Dietary recommendations included substituting refined carbohydrates with whole-grain meals and increasing the consumption of vegetables and other dietary sources rich in fiber.

**Group 2 (n=20):** patients with T2DM treated with Metformin 500 mg/day in addition to nonpharmacological therapy (lifestyle modification) for one month.

**Group 3 (n=68):** patients with T2DM treated with Metformin 500 mg/day plus vitamin B6 300 mg/day in addition to non-pharmacological therapy (lifestyle modification) for one month.

### Outcome measurement

Blood samples (10 ml) were collected from all participants at the beginning and end of the trial. These samples were obtained following a 12- to 14-hour overnight fast while the individuals were in a seated posture between 7:00 and 9:00 AM. The blood samples were tested at the Mesan Center for Diabetes and Endocrinology laboratory, certified and operated by the government. The analysis was conducted on the same day as the blood collection and included the following tests: PLP blood level, fasting plasma glucose (FPG), fasting plasma insulin (FPI), homeostasis model assessment of insulin resistance (HOMA-IR), glycated hemoglobin (HbAlc), indoleamine 2,3-dioxygenase (IDO).

### Statistical analysis

Statistical analyses were conducted using SPSS version 25 for Windows (RRID: SCR 016479) to determine the influence of various variables on the studied parameters. The Shapiro-Wilk test was selected as the normality test. The Kruskal–Wallis test was used to statistically compare means when data were presented as mean ± standard deviation (SD), with a significance level of p<0.05. Differences between pre-and post-treatment outcomes were evaluated using the paired t-test, while unpaired t-tests were utilized to compare changes in patient pre- and post-treatment results between groups 1 and 2. One-way ANOVA was conducted to compare the measured parameters, followed by post-hoc Tukey's test to determine significant differences among the groups. Additionally, the chi-square test was employed to identify significant correlations among demographic variables. To test the hypotheses and confirm relationships, relationships between observed and latent (unobserved) variables were conducted using IBM SPSS Amos 26. For regression (impact) testing, the study depended on the structural model using the structural modeling equation (SEM) approach.

## RESULTS

### Demographic characteristics

**Figure 1:**
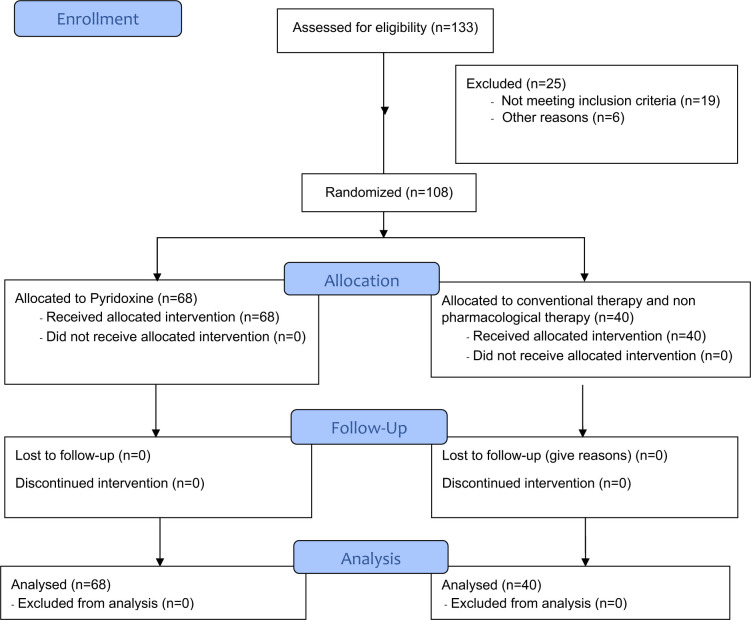
CONSORT Flow Diagram

A total of 108 individuals were involved in the present investigation. The study included 55 men and 53 women (Figure 1). The ages of participants ranged from 30 to 61 years. The mean age for women was 43.4 years with a standard deviation of 6.96 years, while for men, the mean age was 41.5 years with a standard deviation of 10.75 years, as shown in Table 1.

**Table 1:** Demographic characteristics

		Group 1	Group 2	Group 3	Total
**Number of subjects**		**20**	**20**	**68**	**108**
**Gender n (%)**	**Male participants**	11 (55%)	10 (50%)	40 (59%)	55 (51%)
	**Female participants**	9 (45%)	10 (50%)	28 (41%)	53 (49%)
**Age male subjects**	**Mean**	40.4±12.98	41.9±15.66	43.8±17.14	41.5±10.75
	**Range**	30-59	32-61	32-61	30-61
**Age female subjects**	**Mean**	42.8±11.84	43.9±10.36	44.7±12.28	43.4±6.96
	**Range**	32-58	33-60	30-61	30-61

### Comparison between baseline characteristics

An unpaired t-test was used to assess the baseline characteristics. The findings revealed significant differences in PLP, FPG, HbA1c, fasting insulin, HOMA-IR, and IDO. Table 2 shows the mean and standard deviation of these variables in the control and patient groups.

**Table 2:** Baseline characteristics

Parameters	Treatment groups		Control group	
	mean	±SD	mean	±SD
**Pyridoxine blood level (mcg/L)**	22.7	1.022	39.5	2.158
**Fasting plasma glucose (mg/dL)**	138.42	5.555	83.53	4.73
**Hemoglobin A1C (%)**	6.938	0.092	5.12	0.064
**Fasting insulin (mIU/L)**	18.54	1.888	11.14	1.225
**Homeostasis model assessment of insulin resistance (HOMA-IR)**	6.337	0.604	2.298	0.712
**Indoleamine 2,3-dioxygenase**	22.62	2.54	18.40	2.77

### Effect of interventions on pyridoxine concentration

Table 3 presents the statistically significant (p<0.05) increase in PLP observed in all study groups at the end of the four weeks compared to their respective baseline readings. The increase in PLP was significantly greater in the Metformin and Metformin + vitamin B6 groups (p<0.05) than in the control group. There was a significant difference in the increase in PLP between the Metformin + vitamin B6 and Metformin groups when analyzing the change in study groups without including the control group (Table 3).

**Table 3:** Effect of study treatment on pyridoxine blood level

Groups		G 1		G 2		G 3		p-value
Parameter		mean	±SD	mean	±SD	mean	±SD	
**PLP**	**Baseline**	23.21	4.02	22.87	6.13	23.14	5.6	p<0.05
	**4 weeks**	29.14	5.66	31.3^2^*	4.24	40.77^**^	4.2	
	**p-value**	p>0.05		p<0.05		p<0.001		
	**Δ PLP**	5.93	0.21	8.45^a^	0.12	17.63^ab^	0.17	
	**Δ PLP %**	25.55%		36.95%		76.19%		

PLP: pyridoxal-5-phosphate. Δ PLP: change in pyridoxal-5-phosphate, *statistically significant, **highly significant p<0.001

### Effect of interventions on fasting plasma glucose and HbA1c

There was a significant decrease in FPG and HbA1c levels among all study groups after four weeks compared to their respective baseline data (Table 4). The Metformin and Metformin + vitamin B6 groups exhibited significantly greater reductions in FPG and HbA1c levels compared to the control group (p<0.05).

**Table 4:** Effect of study treatment on FPG and HbA1c

Groups		G 1		G 2		G 3		p-value after 4 weeks
parameters		mean	±SD	mean	±SD	mean	±SD	
**FPG**	**Baseline**	130.12	5.42	135.55	4.34	142.22	4.35	p<0.05
	**4 weeks**	111.25^*^	4.97	102.21^*^	4.28	89.75^**^	3.88	
	**p-value**	p<0.05		p<0.05		p<0.001		
	**Δ FPG**	-18.87	1.22	-33.34^a^	1.12	-52.47^a^	1.14	
	**Δ FPG %**	-14.50%		-24.60%		-36.89%		
**HbA1c**	**Baseline**	6.32	0.084	6.75	0.092	7.13	0.091	p<0.05
	**4 weeks**	6.1^*^	0.081	6.01^*^	0.091	5.94^**^	0.089	
	**p-value**	p<0.05		p<0.05		p<0.001		
	**ΔHbA1c**	-0.22	0.022	-0.74^a^	0.017	-1.19^a^	0.011	
	**Δ HbA1c %**	-3.48%		-10.96%		-16.69%		

FPG: fasting plasma glucose, Δ FPG: changes in fasting plasma glucose *statistically significant, **highly significant p<0.001

### Effect of treatment on fasting insulin and HOMA-IR

As demonstrated in Table 5, all study groups showed a significant decrease in fasting insulin and HOMA-IR levels after four weeks (p<0.05) compared to baseline data. The Metformin and Metformin + vitamin B6 groups had significantly lower fasting insulin and HOMA-IR compared to the control group (p<0.05).

**Table 5:** Effect of study treatment on fasting insulin and HOMA-IR

Groups		G 1		G 2		G 3		p-value after 4 weeks
parameters		mean	±SD	mean	±SD	mean	±SD	
**Fasting insulin (FI)**	**Baseline**	12.44	1.17	12.27	1.22	12.01	1.11	p<0.05
	**4 weeks**	11.09^*^	1.13	10.12^*^	1.07	8.41^**^	0.76	
	**p-value**	p<0.05		p<0.05		p<0.001		
	**ΔFI**	-1.35	0.14	-2.15^a^	0.17	-3.6^a^	0.19	
	**ΔFI %**	-10.85%		-17.52%		-29.98%		
**HOMA-IR**	**Baseline**	3.997	0.34	4.107	0.35	4.217	0.37	p<0.05
	**4 weeks**	3.046^*^	0.33	2.554^*^	0.38	1.864^**^	0.33	
	**p-value**	p<0.05		p<0.05		p<0.001		
	**ΔHOMA-IR**	-0.950	0.07	-1.553^a^	0.02	-2.354^a^	0.01	
	**ΔHOMA-IR %**	-23.77%		-37.81%		-55.82%		

FI: fasting insulin, *statistically significant, **highly significant p<0.001

### Effect of interventions on indoleamine 2,3-dioxygenase

Table 6 displays a significant reduction in IDO levels among all research groups after four weeks, compared to their initial values (p<0.05). Compared to the control group, the reduction in IDO was significantly greater in the Metformin and Metformin plus vitamin B6 groups (p<0.05) at week 4.

**Table 6:** Effect of study treatment on Indoleamine 2,3-dioxygenase

Groups		G 1		G 2		G 3		p-value after 4 weeks
parameters		mean	±SD	mean	±SD	mean	±SD	
**IDO**	**Baseline**	21.11	2.25	22.23	1.38	22.97	1.34	p<0.05
	**4 weeks**	19.48	2.34	19.01^*^	1.36	18.11^**^	1.27	
	**p-value**	p>0.05		p<0.05		p<0.001		
	**ΔIDO**	-1.63	0.23	-3.22^a^	0.28	-4.86^a^	0.17	
	**ΔIDO %**	-7.72%		-14.48%		-21.16%		

* statistically significant, **highly significant p<0.001

### Effect of PLP on FPG

The results shown in Table 7 indicate that PLP had an effect on FPG. The analysis revealed a significant negative regression association between PLP and FPG. The standard impact coefficient was calculated at -.876, and the critical ratio (CR) was -4.475, exceeding the threshold of ±1.96. The model consisting of PLP accounts for a significant proportion of the variation in FPG, specifically 77%, whereas the remaining 23% can be attributed to unaccounted factors in the statistical model.

**Table 7:** Impact of PLP on the studied parameters

Regression path	Standardized regression	The standard error (S.E.)	Critical ratio (C.R.)	R^2^	p-value
**FPG <--- PLP**	-.876	.001	-4.475	.767	***
**HbA1C <--- PLP**	-.786	.000	-4.870	.618	***
**FI <--- PLP**	-.731	.000	-3.149	.535	***
**HOMA-IR <--- PLP**	-.692	.000	-3.908	.478	***
**IDO <--- PLP**	-.694	.000	-3.580	.481	***

*** The probability value is less than 0.001, FPG: fasting plasma glucose, PLP: Pyridoxine blood level 'pyridoxal 5-phosphate', HbA1C: Hemoglobin A1C, FI: fasting insulin, HOMA-IR: Homeostasis model assessment of insulin resistance, IDO: Indoleamine 2,3-dioxygenase

### Impact of PLP on HbA1C

PLP had a significant effect on HbA1C levels (Table 7). The analysis revealed a substantial negative regression association between PLP and HbA1C, as evidenced by the standard impact coefficient value of -.786 and CR of -4.870, surpassing the threshold of ±1.96. Table 7 indicates that PLP accounted for 62% of the variability in HbA1C, while the remaining 38% was attributable to unaccounted factors within the statistical model.

### Impact of PLP on fasting insulin

The findings presented in Table 7 indicate a statistically significant association between PLP and fasting insulin levels. There was a significant and negative regression relationship between PLP and fasting insulin. This was supported by the standard impact coefficient of -.731 and the CR of -3.149, beyond the threshold of ±1.96. A substantial percentage of the observed variance in fasting insulin levels, namely 53%, may be attributed to the influence of PLP. The remaining 47% of the components may be attributed to unidentified variables inside the statistical model.

### Impact of PLP on HOMA-IR

PLP had a significant impact on HOMA-IR. The analysis revealed a significant negative regression association between PLP and HOMA-IR (Table 7). This was evidenced by the common impact coefficient value of -.692 and the CR of -3.908, which exceeded the threshold of ±1.96. PLP accounted for 48% of the variance in HOMA-IR, while the remaining 52% was attributable to unaccounted factors within the statistical model.

### Impact of PLP on indoleamine 2,3-dioxygenase

PLP had a significant effect on IDO, revealing a statistically significant negative regression relationship between PLP and IDO. This was evidenced by the standard impact coefficient value of -.694 and the CR value of -3.580, which exceeded the threshold of ±1.96. Table 7 indicates that PLP accounted for 48% of the variance in IDO, while the remaining proportion (52%) was attributable to unaccounted factors within the statistical model.

## DISCUSSION

The findings of this trial indicated a significantly positive effect of using pyridoxine as an additional therapy with Metformin on blood glucose levels and other factors examined in the research. Statistically significant reductions in fasting plasma glucose and HbA1c were seen in groups G2 and G3 during a 4-week treatment period compared to the patients in the control group (G1). Significant reductions in fasting serum insulin and HOMA-IR levels were found in groups G2 and G3 (p<0.05). Research has demonstrated that the inadequacy of vitamin B6 significantly impacts the development of glucose intolerance [15, 23, 24]. PLP, the biologically active coenzyme form of vitamin B6, contributes to the processes of gluconeogenesis and glycogenolysis via its involvement in transaminase activities and glycogen phosphorylation [25]. The results of this study align with prior research that has shown decreased levels of PLP in individuals diagnosed with T2DM compared to a control cohort [26]. Many previous studies have investigated the impact of PLP on FPG levels. In a comparative randomized controlled study, Hlais *et al*. documented a decrease in FPG levels after the injection of PLP. The findings of their study align with our own, suggesting that the use of PLP enhances glucose metabolism [27]. The study conducted by Khobrani *et al*. examined the effects of PLP supplementation on glycemic regulation among individuals diagnosed with T2DM. In this study, the administration of PLP supplements showed a significant decrease in HbA1c levels, indicating improved glycemic control. According to the study findings, the supplementation of PLP can be a beneficial supplementary therapy for those diagnosed with type 2 diabetes [28]. In 2021, Haidari *et al*. did a comprehensive review and meta-analysis to assess the literature concerning the association between vitamin B6 levels and glycemic indices. There exists a significant association between decreased levels of PLP and elevated fasting insulin and HOMA-IR readings, indicating a distinct correlation between inadequate vitamin B6 levels and insulin resistance in individuals with type 2 diabetes [29]. Mascolo and Vern conducted a review to explore the correlation between vitamin B6 status and molecular mechanisms in diabetes. Their findings revealed a positive association between vitamin B6 levels and IDO activity, suggesting that increased concentrations of vitamin B6 may enhance the functioning of IDO. Recent studies indicate that PLP supplements may potentially lead to a reduction in IDO activity [13]. The study's results suggest that the provision of pyridoxine supplements to individuals with diabetes is a very cost-effective strategy for mitigating the risk of developing diabetes. The efficacy of pyridoxine in the prevention of T2DM is supported by the findings of a recent clinical study. The study showed that regular consumption of pyridoxine by patients with diabetes resulted in a decreased rate of overt diabetes development. It is posited that the present research findings may provide valuable insights to healthcare officials in optimizing the allocation of financial resources and devising comprehensive national programs aimed at preventing diabetes. Despite this being one of the largest randomized controlled trials of a supplemental agent carried out to date for the enhancement of glycemic indicators, one limitation of the research is that only 108 individuals were investigated. Consequently, there might not have been enough power to identify disparities among non-pharmacological, Metformin, and pyridoxine-treated groups for the diabetes-related parameters examined. Because the research period was only one month, the study did not investigate the effectiveness of pyridoxine over the long term, which is essential for chronic illnesses such as diabetes. The enrollment of newly diagnosed patients, utilization of thorough diabetic evaluations, and outstanding subject adherence are all strong points of this trial.

## CONCLUSION

Several key conclusions can be drawn based on the results obtained from this study. The addition of vitamin B6 to Metformin treatment had a positive impact on blood glucose levels in individuals with T2DM, resulting in a reduction in blood glucose levels. Vitamin B6 deficiency is one of the causes of diabetes, as it is a catalyst for the metabolism of carbohydrates. Diabetes leads to a deficiency in vitamin B6 in the body due to its increased use in metabolic processes to regulate increased blood glucose levels.
